# A high-resolution description of β_1_-adrenergic receptor functional dynamics and allosteric coupling from backbone NMR

**DOI:** 10.1038/s41467-020-15864-y

**Published:** 2020-05-05

**Authors:** Anne Grahl, Layara Akemi Abiko, Shin Isogai, Timothy Sharpe, Stephan Grzesiek

**Affiliations:** 1 Focal Area Structural Biology and Biophysics, Biozentrum, University of Basel, 4056 Basel, Switzerland; 2 Biophysics Core Facility, Biozentrum, University of Basel, 4056 Basel, Switzerland

**Keywords:** Biochemistry, NMR spectroscopy

## Abstract

Signal transmission and regulation of G-protein-coupled receptors (GPCRs) by extra- and intracellular ligands occurs via modulation of complex conformational equilibria, but their exact kinetic details and underlying atomic mechanisms are unknown. Here we quantified these dynamic equilibria in the β_1_-adrenergic receptor in its apo form and seven ligand complexes using ^1^H/^15^N NMR spectroscopy. We observe three major exchanging conformations: an inactive conformation (*C*_i_), a preactive conformation (*C*_p_) and an active conformation (*C*_a_), which becomes fully populated in a ternary complex with a G protein mimicking nanobody. The *C*_i_ ↔ *C*_p_ exchange occurs on the microsecond scale, the *C*_p_ ↔ *C*_a_ exchange is slower than ~5 ms and only occurs in the presence of two highly conserved tyrosines (Y^5.58^, Y^7.53^), which stabilize the active conformation of TM6. The *C*_p_→*C*_a_ chemical shift changes indicate a pivoting motion of the entire TM6 that couples the effector site to the orthosteric ligand pocket.

## Abstract (web summary)

Signal transmission and regulation of G-protein-coupled receptors (GPCRs) by ligands occurs via modulation of complex conformational equilibria. Here authors quantify these equilibria and their dynamics in the β1-adrenergic receptor in its apo form and seven ligand complexes using NMR.

## Introduction

G protein-coupled receptors (GPCRs) are transmembrane signal transducers, which convert the extracellular binding of ligands to specific intracellular responses via G protein, arrestin and other pathways. As regulators of crucial physiological processes, the more than 800 GPCRs within the human proteome have long been prime drug targets^1,2^. Advances in their structural stabilization by protein engineering, in crystallization and electron microscopic techniques have led to a surge of available crystal and cryo-EM structures in the last decade^3–10^. These frozen snapshots range from inactive forms in antagonist complexes to various active forms in complexes with agonists, G protein, G protein-mimicking antibodies and arrestin.

Strikingly, the crystal structures of antagonist- or agonist-bound receptors are very similar^11^, although their conformational states should encode their functional difference. Substantial changes in crystal structures are only observed for ternary agonist·receptor complexes with a G protein or a G protein-mimicking nanobody bound to the intracellular effector side, in which transmembrane helices 5 and 6 (TM5,6) move by up to 14 Å outward from the transmembrane 7-helix bundle (see below)^12–14^.

In contrast, evidence from EPR, NMR, fluorescence spectroscopic data and molecular dynamics simulations indicates that GPCRs are highly dynamic and sample several conformations in any particular functional state^5,15–23^. Thus signal transmission in the receptor must occur via shifts in dynamic equilibria and the key to understanding GPCR function is the accurate and precise description of its functional motions at atomic resolution—simultaneously at many sites throughout the receptor. No such comprehensive description of GPCR dynamics exists at present.

In principle, NMR spectroscopy can provide precise dynamical information for any atom site with a magnetically active nucleus by the analysis of relaxation parameters. In practice, NMR observation of GPCRs is strongly limited in sensitivity and resolution by intrinsically broad line widths and by difficulties in isotope labeling in higher eukaryotic cells^24^. For these reasons, NMR observations of GPCR dynamics have been confined mostly to chemical shift changes induced by different ligands and to the qualitative description of line broadening effects in selectively ^13^C-labeled side chain methyl groups^16,17,19,25–27^ or ^19^F-labeled side chains^15,21,28,29^.

The NMR detection of main chain atomic nuclei is less sensitive, but in particular ^1^H-^15^N resonances have the advantage of directly reporting on backbone and H-bond conformational changes with functional relevance. Despite early efforts^30^, such ^1^H-^15^N backbone resonances have only recently been detected at high resolution^20,23,31^. In particular, we observed well resolved ^1^H-^15^N resonances in a thermostabilized mutant of the turkey β_1_-adrenergic receptor (β_1_AR), which had been prepared in detergent-solubilized, isotope-labeled form from insect cells^20,32^. The changes of the ^1^H-^15^N chemical shifts in response to various ligands gave evidence of induced structural changes throughout the receptor, but did not provide information on the timescales of the functional equilibria, their underlying mechanics and the effect of thermostabilizing mutations on these equilibria.

**Fig. 1:**
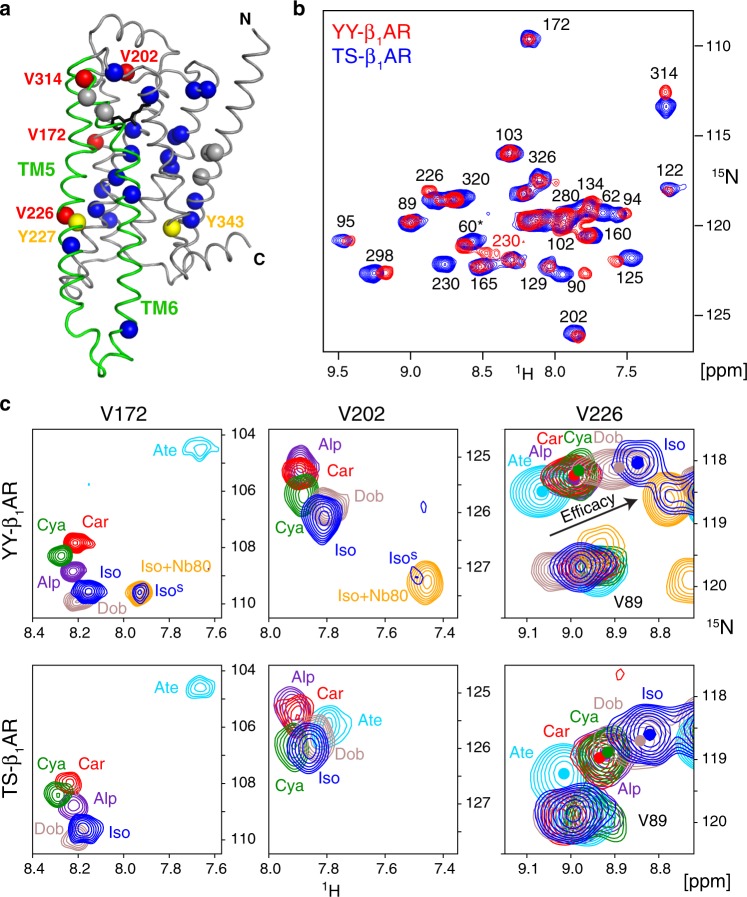
Ligand-induced ^1^H-^15^N chemical shift changes in the YY-β_1_AR and TS-β_1_AR constructs. **a** Crystal structure of β_1_AR in complex with isoprenaline (2Y03). The protein backbone and isoprenaline are shown in ribbon and stick, respectively, with TM5 and TM6 highlighted in green. Valines are shown as spheres (blue: assigned; gray: not assigned; red: particularly strong chemical shift changes in response to ligands). Tyrosines 227^5.58^ and 343^7.53^ are depicted as yellow spheres. **b** ^1^H-^15^N TROSY spectra of ^15^N-valine-labeled TS-β_1_AR (blue) and YY-β_1_AR (red) in decyl maltoside micelles in the presence of 1 mM isoprenaline. Resonances are marked with assignment information. The assignment of V60 is tentative and marked by an asterisk. **c** Comparison of V172^4.59^, V202^ECL2^ and V226^5.57 1^H-^15^N TROSY resonances of YY-β_1_AR and TS-β_1_AR in orthosteric binary and ternary isoprenaline•Nb80 complexes. Resonances of the YY-β_1_AR•isoprenaline complex (blue) marked by superscript ‘s’ correspond to a second conformation, which coincides with the active conformation in the ternary complex (orange). The arrow indicates the approximate linear correlation between ^1^H and ^15^N chemical shifts of V226^5.57^ in various ligand-bound states and ligand efficacy (see also Supplementary Fig. 2).

Here we obtain a comprehensive quantitative description of the dynamics of the β_1_AR in its apo form, in response to six ligands ranging from inverse agonists to agonists, as well as in an agonist/G protein mimetic nanobody (Nb80) complex from precise measurements of ^15^N NMR relaxation rates at 14 backbone amide sites. We compare this information for an ultrastable β_1_AR mutant (TS-β_1_AR, melting temperature *T*_m_ = 59 °C), which is deficient in G protein activation, and the TS-β_1_AR_A227Y/L343Y_ double mutant (named YY-β_1_AR in the following), which recovers G protein activation by reintroducing the conserved tyrosines Y^5.58^ and Y^7.53^ [the superscript corresponds to Ballesteros–Weinstein numbering^33^] in TM5 and TM7 (Fig. 1a) at the expense of stability (*T*_m_ = 48 °C)^20^. The results reveal highly similar fast equilibria on the micro- to millisecond timescale between inactive (*C*_i_) and preactive (*C*_p_) conformations throughout the receptor for both mutants that correlate to ligand efficacy and ligand affinity. These fast equilibria can be described for all observations to high precision by a simple linear function of two parameters. In contrast, a slow (>5 ms) equilibrium towards the active conformation *C*_a_ occurs only for the YY-β_1_AR construct, which is rationalized by the formation of a water-mediated hydrogen bond bridge between Y^5.58^ and Y^7.53^. This bridge stabilizes the active conformation of TM6 in its the swung-out position from the helix bundle. The chemical shift data in *C*_p_ and *C*_a_ show a rearrangement of the extracellular binding pocket, which can be rationalized by a pivoting motion of TM6 and explains ligand affinity variations and antagonistic function of antagonists with large hydrophobic head group substitutions.

## Results

### Observation of distinct fast and slow timescale equilibria

We had previously observed^20^ that the ^1^H-^15^N resonances of many valines (Fig. 1a) of the ^15^N-valine-labeled, ultrastable TS-β_1_AR showed very high correlations to particular ligand properties (see below) such as ligand chemistry (V172^4.56^ located close to the ligand head group), efficacy for G protein activation (V226^5.57^ at the intracellular end of TM5), and ligand affinity (V314^6.59^ at the extracellular end of TM6). In an effort to understand the structural basis why the two tyrosines Y^5.58^ and Y^7.53^ restore G protein activation in YY-β_1_AR, we have now systematically compared the spectra of all binary orthosteric ligand complexes of both ^15^N-valine-labeled YY-β_1_AR and TS-β_1_AR constructs. Their high spectral similarity for all ligand complexes and very similar continuous shifts of resonance lines according to various ligand properties (Fig. 1b, c, Supplementary Figs. 1 and 2) indicate that the average conformations of both receptor constructs and their responses to ligands are highly similar.

An exception is observed for the complex of YY-β_1_AR with the agonist isoprenaline. This complex shows a main set of resonances with similar positions as the TS-β_1_AR•isoprenaline complex, but also a previously unnoted, second set of weak resonances corresponding to about 20% population of a further state, which is clearly detectable for residues V172^4.56^, V202^ECL2^ (Fig. 1c), V314^6.59^ and further residues around the ligand pocket (see below). These minor ^1^H-^15^N resonances coincide with the resonances of the respective YY-β_1_AR residues in the ternary complex with isoprenaline and the G protein-mimicking nanobody Nb80. This indicates that for the observed region around the ligand pocket the isoprenaline-bound receptor is in slow (>5 ms) conformational exchange with the ‘active’ conformation (*C*_a_) that becomes 100% populated upon ternary complex formation with the G protein-mimicking nanobody Nb80. We have recently shown that the population of this active conformation in the absence of Nb80 can be increased by the application of pressure^34^ giving evidence that its volume is about 100 Å^3^ smaller than that of the main conformation. Apparently, the main ‘preactive’ conformation (*C*_p_) of the binary isoprenaline•YY-β_1_AR complex is primed to undergo a well-defined conformational switch to the active conformation *C*_a_—at least in the surroundings of the ligand at the extracellular side—even in the absence of an effector protein.

Taken together these findings indicate that YY-β_1_AR is in a fast equilibrium on the chemical shift timescale of micro- to milliseconds for all non-agonist complexes and the main set of resonances of the complex with the agonist isoprenaline. However, this latter preactive state also experiences a second equilibrium on a slow supra-millisecond timescale towards the active state in the vicinity of the ligand. We discuss first the fast equilibrium observed by the main resonances of all binary orthosteric ligand complexes and later address the slow equilibrium of the agonist complex.

The high similarity of both receptor constructs is evident from the almost identical ^1^H^N^ and ^15^N positions of the main resonances of V172^4.56^, V226^5.57^ and V314^6.59^ (Pearson *r* > 0.90 in all cases, Supplementary Fig. 2), which show the strongest variations in response to ligands (see below). Thus the average conformations at the ligand-binding pocket, the intracellular effector binding site, and the extracellular side must be very similar in both β_1_AR forms and respond similarly to different ligand properties. In particular, the ^1^H-^15^N resonances of V226^5.57^ and V314^6.59^ of YY-β_1_AR fall on almost identical single lines in response to the different ligands (Fig. 1c, Supplementary Fig. 2), yielding similar high correlations to the efficacy for G protein activation (V226^5.57^, *r* = 0.91, Supplementary Fig. 2) and ligand affinity (V314^6.59^, *r* = 0.94, see below) as the TS-β_1_AR construct^20^. This single-line behavior for V226^5.57^ and V314^6.59^ indicates that the average conformations of the fast equilibrium at the extracellular ligand binding pocket and at the intracellular effector site follow a continuous path in response to the various ligands. No such continuous path is observed for the ^1^H-^15^N resonances of V172^4.56^ close to the ligand head group, which scatter within the ^1^H-^15^N plane (Fig. 1c), indicating that the average conformations at this location vary in a more complicated manner according to the details of the chemical structure of the ligand.

### Determination of receptor dynamics from ^15^N relaxation rates

**Fig. 2:**
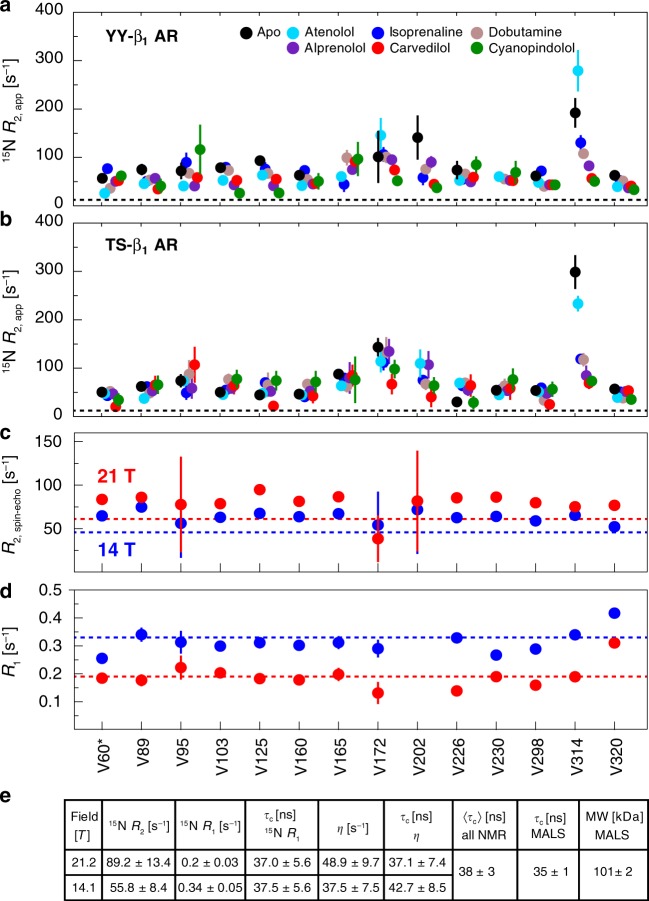
^15^N relaxation rates of YY-β_1_AR and TS-β_1_AR. **a**, **b** Residue-specific ^15^N apparent transverse relaxation rates *R*_2,app_ of ^15^N-valine YY-β_1_AR and TS-β_1_AR derived from line shape fitting of the ^1^H-^15^N TROSY spectra in the apo form and orthosteric binary complexes recorded at 304 K and 21 T (YY-β_1_AR) or 19 T (TS-β_1_AR). Due to limited stability, the apo form of YY-β_1_AR was recorded at 294 K. Data are color-coded according to the ligand. The dashed lines represent theoretical TROSY ^15^N *R*_2_ rates for a rotational correlation time *τ*_c_ of 38 ns. **c**, **d** Residue-specific ^15^N spin-echo transverse (*R*_2,spin-echo_) and longitudinal (*R*_1_) rates of alprenolol-bound ^15^N-valine TS-β_1_AR recorded at 304 K and 21 T (red) or 14 T (blue). Dashed lines represent theoretical *R*_2_ and *R*_1_ rates. The assignment of V60 is tentative and marked by an asterisk. Numerical data for **a**–**d** are provided in Supplementary Data 3. Error values were derived from Monte Carlo fitting as indicated in Methods. **e** Average ^15^N relaxation rates obtained from 1D experiments on ^2^H (~60%)/^15^N-labeled, alprenolol-bound TS-β_1_AR (Supplementary Fig. 3), molecular weight by MALS, and rotational correlation times τ_c_ derived from NMR and MALS.

To characterize the timescale of receptor motions, we determined various ^15^N relaxation parameters. Apparent TROSY transverse relaxation rates *R*_2,app_(^15^N_x_^1^H_β_) were calculated by time-domain fitting of the main resonances for all ligand complexes (Fig. 2a, b). For both mutants, most residues have *R*_2,app_ values around ~50 s^−1^, but strong increases up to ~250 s^−1^ occur for residues V172^4.56^ as well as V314^6.59^ and the close-by V202^ECL2^ at the extracellular ligand entry site with the atenolol complex and the apo form having the largest rates. These increased *R*_2,app_ rates must be caused by conformational exchange on the chemical shift timescale of micro- to milliseconds. The highly similar transverse relaxation rates across the entire receptor indicate that both mutants have very similar micro- to millisecond motions in all ligand complexes at the positions of all observed amino acids. It should be noted that contributions from the *B*_0_ field inhomogeneity to *R*_2,app_ are smaller than 1 s^−1^ based on the quality of the spectrometer shimming and are therefore negligible.

To characterize the receptor behavior on the nanosecond timescale and to obtain estimates on exchange-free *R*_2_ relaxation rates, several further ^15^N relaxation rates were determined by conventional, less sensitive relaxation experiments on ^15^N/^2^H(~60%)-labeled TS-β_1_AR as well as on ^15^N-valine-labeled TS-β_1_AR, both in complex with alprenolol (Fig. 2, Supplementary Fig. 3). Besides residue V320^ECL3^, the longitudinal ^15^N *R*_1_ rates (Fig. 2d) are uniform with average values of 0.34 s^−^^1^ at 14 T and 0.20 s^−1^ at 21 T in agreement with the expected field dependence of the slow tumbling limit. The isotropic rotational correlation times *τ*_c_ of 38 ± 3 ns derived from these *R*_1_ rates and additionally measured dipolar-coupling/CSA cross-correlation rates *η* agree very well with the value of 35 ± 1 ns expected from the 101-kDa micellar mass determined in a multi-angle light-scattering (SEC-MALS) experiment (Fig. 2e). Thus β_1_AR rotates at the same speed as the entire micelle and a significant additional nanosecond motion of the GPCR relative to the detergent can be excluded. Interestingly, V320 is located in extracellular loop 3 and its higher *R*_1_ rate indicates increased nanosecond mobility in this region.

*R*_2_(^15^N_*x*_) rates were also determined by a conventional spin-echo experiment^35^ with a CPMG ^15^N 180°-pulse spacing of 0.5 ms (*ν*_CP_ = 2 kHz). Lower effective field strengths *ν*_CP_ were impractical due to the required ^1^H decoupling and the short ^15^N_*x*_ *T*_2_ time of 10–20 ms. Similar to the *R*_1_ rates, the *R*_2_(^15^N_*x*_) rates are quite uniform (~60 s^−1^ at 14 T and ~80 s^−1^ at 21 T, Fig. 2c) for all detected TS-β_1_AR ^15^N-valine residues and agree well with the average values determined on the ^15^N/^2^H(~60%)-labeled TS-β_1_AR (Fig. 2e). However, these *R*_2_(^15^N_*x*_) rates are considerably larger than the exchange-free *R*_2,0_(^15^N_*x*_) rates of 46 s^−1^ at 14 T (61 s^−1^ at 21 T) calculated for a spherical molecule tumbling with a *τ*_c_ of 38 ns. The resulting values for the exchange contribution *R*_ex_ (=*R*_2_ − *R*_2,0_) of ~10–30 s^−1^ corroborate the presence of micro- to millisecond motions for many residues of the receptor.

Remarkably, the exchange contributions *R*_ex,app_(^15^N_*x*_^1^H_β_) = *R*_2,app_(^15^N_*x*_^1^H_β_) − *R*_2,0_(^15^N_*x*_^1^H_β_) determined from the TROSY line shape analysis (Fig. 2b) are significantly larger and vary much more than the *R*_ex_(^15^N_*x*_) values observed in the spin-echo experiments. Thus, the 2 kHz CPMG field of the *R*_2_(^15^N_*x*_) experiment quenches part of the exchange broadening. This effect is particularly pronounced for V314^6.59^, which shows very strong ^15^N line broadening in the TROSY, but *R*_2_(^15^N_*x*_) values identical to other residues in the spin-echo. From this quenching, we estimate that the exchange rates are on the order of 10^3^–10^4^ s^−1^.

### Effects of ligand meta and ortho head group substitutions

**Fig. 3:**
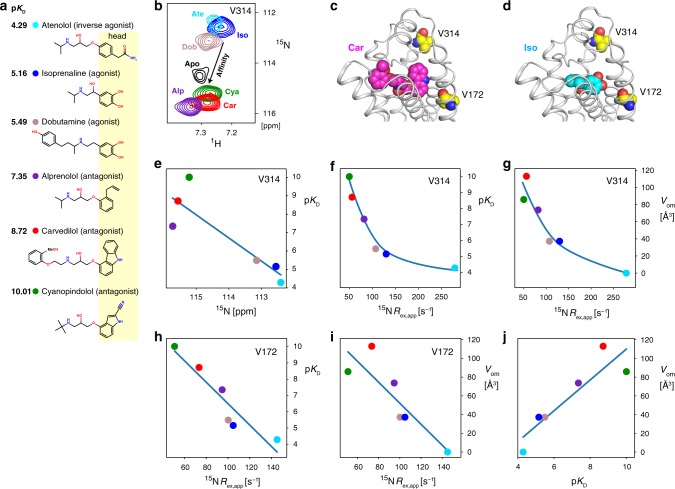
Correlation of structure and dynamics around the ligand-binding pocket to ligand head group volume. **a** Chemical structures of the ligands used in the experiments. Ligand affinities^58^ are indicated as p*K*_D_ values. **b** Selected region from ^1^H-^15^N TROSYs showing the variation of the V314^6.59 1^H-^15^N resonance in the apo form and orthosteric binary complexes of YY-β_1_AR at 21 T. Spectra are color-coded and marked according to the ligand. Due to limited stability, the apo form of YY-β_1_AR was recorded at 294 K. The arrow indicates the approximate linear correlation between ^1^H and ^15^N chemical shifts of V314^6.59^ in various ligand-bound states and ligand affinity (see also Supplementary Fig. 2). **c**, **d** Partial views of the β_1_AR crystal structures in complex with carvedilol (4AMJ) and isoprenaline (2Y03) showing V172 and V314 (yellow) in the vicinity of the ligand (magenta, cyan). **e**–**j** Correlations of spectral and ligand properties. Lines are drawn to guide the eye. **e** p*K*_D_, V314^6.59 15^N chemical shift. **f** p*K*_D_, V314^6.59 15^N *R*_ex, app_. **g** Volume of ligand head group ortho, meta substitutions (*V*_om_), V314^6.59 15^N *R*_ex, app_. **h** p*K*_D_, V172^4.56 15^N *R*_ex,app_. **i** *V*_om_, V172^4.56 15^N *R*_ex, app_. **j** *V*_om_, p*K*_D_. Numerical data for **e**–**j** are provided in Supplementary Data 1, 2.

It is remarkable that the ^15^N chemical shift of V314^6.59^ at the entrance to the orthosteric ligand-binding pocket varies over a large range from 116 to 112 ppm in different binary receptor complexes and correlates strongly to the ligand affinity p*K*_D_ (Fig. 3b, e). These chemical shifts are unusual as compared to most other residues (range ~118–126 ppm, Fig. 1b) and indicate an unusual backbone geometry (see below). The strongest deviations in chemical shift occur for the low affinity ligands isoprenaline, dobutamine (both agonists) and atenolol (inverse agonist). The apo form is located between these low affinity ligands and the less shifted high-affinity ligands alprenolol, cyanopindolol and carvedilol (all antagonists). Furthermore, also the exchange broadening contributions *R*_ex,app_(^15^N_*x*_^1^H_β_) correlate inversely to p*K*_D_ (Fig. 3f) with the low affinity ligands showing the largest broadening. A similar inverse correlation is observed for the *R*_ex,app_(^15^N_*x*_^1^H_β_) of V172^4.56^ close to the ligand head group (Fig. 3h), albeit no strong correlation exists between p*K*_D_ and its ^15^N chemical shift (Fig. 1c).

Apparently, ligands with high affinity reduce exchange broadening. In search for a rationale for this behavior, we determined the volume of the meta and ortho substitutions *V*_om_ on the aromatic ring of the ligand head group (Supplementary Data 1, see below), as these substitutions point into a cavity of the ligand pocket between TM5 and TM6 in the direction of residue V314^6.59^ (Fig. 3c, d) and their presence may reduce mobility of adjacent residues. Indeed, *V*_om_ anti-correlates with the *R*_ex,app_(^15^N_*x*_^1^H_β_) values of V314^6.59^ (*r* = −0.88) and V172^4.56^ (*r* = −0.87) (Fig. 3g, i). Their high *R*_ex,app_(^15^N_*x*_^1^H_β_) values in the apo form (Fig. 2a) agree with this observation. Obviously, then also a high positive correlation must exist between *V*_om_ and p*K*_D_ (Fig. 3j, *r* = 0.90). Thus the cause for the line broadening is conformational freedom due to a remaining void in the ligand pocket, which is strongly reduced when large meta and ortho substitutions of the ligand head group are introduced into this cavity. These substitutions increase the contacts to the receptor and thereby affinity. The substitutions of most high-affinity ligands are hydrophobic, which may in part compensate for the loss in entropy of the receptor by the burial of their hydrophobic surface.

### Two parameters describe fast-time-scale receptor behavior

The single-line behavior of the V314^6.59^ and V226^5.57 1^H-^15^N main resonances in response to orthosteric ligands shows that the average receptor conformations within the fast equilibrium follow a continuous path at the ligand entrance pocket and at the G protein effector site, whereas no such continuous path is observed for the ^1^H-^15^N resonances and conformation of V172^4.56^ close to the ligand head group. Notably, the observation of single resonances for all these residues implies that any averaging over subconformations has occurred on a timescale faster than their chemical shift variations (micro- to milliseconds). Thus the receptor is in an approximate equilibrium up to this timescale. Apparently, these fast-time-scale average conformations are almost identical for both receptor mutants. Only on the timescale slower than about 5 ms and only in its agonist-bound form, their behavior differs and the YY-β_1_AR mutant undergoes a further transition to the active conformation as evident from the second set of weak resonances.

**Fig. 4:**
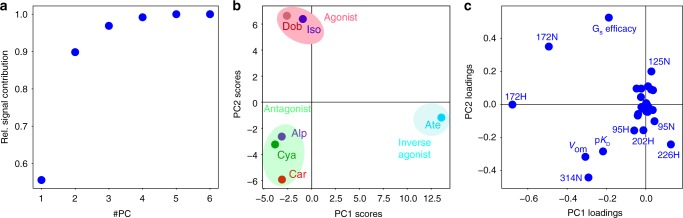
PCA of chemical shift and biochemical data of YY-β_1_AR binary complexes. The PCA was carried out on a total of 186 chemical shifts and biochemical data of the YY-β_1_AR complexes with orthosteric ligands. For the isoprenaline complex, the chemical shifts of the major (preactive) conformation were used. **a** Relative contribution of the principal components to the total observed signal. **b** Scores plot of the first two principal components (PC1 and PC2). Agonists, antagonists and inverse agonist cluster in different regions. **c** Loading plot of the first two principal components. Source data are provided as a Source Data file.

We asked whether these fast-timescale conformational averages as observed by their chemical shifts and other biochemical data could be combined into a single quantitative description of the inactive/preactive receptor. The quantitative data comprise 14 ^1^H-^15^N chemical shift pairs of the valines detected in all 6 orthosteric ligand complexes (Supplementary Data 2), the ligand p*K*_D_s, their efficacies for the G_s_ pathway, as well as the combined volume of the ortho and meta substitutions of the ligand benzene head group (*V*_om_, Supplementary Data 1). A principal component analysis (PCA) of these 31 × 6 observations revealed that 90% of the data could be explained by only two principal components (Fig. 4a), suggesting that a two-dimensional model captures most of the observed receptor behavior within the fast-timescale equilibrium.

The scores of the first two PCA components (Fig. 4b) very clearly separate the different ligand complexes into three distinct clusters according to agonist, antagonist and inverse agonist pharmacology. The main contributors to this separation are evident from the loadings plot (Fig. 4c). Obviously, G_s_ efficacy, *V*_om_ and p*K*_D_ have strong distinguishing power. Similarly strong distinguishing contributions arise from the chemical shifts of V172^4.56^-^1^H, V172^4.56^-^15^N, V314^6.59^-^15^N, V226^5.57^-^1^H and V125^3.36^-^15^N, whereas the other chemical shifts give smaller contributions. The loadings plot also reveals the correlation between the different observations. As expected, p*K*_D_, *V*_om_ and V314^6.59^-^15^N and similarly G_s_ efficacy and V226^5.57^—are almost collinear, whereas G_s_ efficacy and p*K*_D_ or V314^6.59^-^15^N and V226^5.57^-^1^H are almost orthogonal. Interestingly, V172^4.56^-^1^H and V172^4.56^-^15^N are linearly independent and not collinear to either p*K*_D_ or G_s_ efficacy. Thus the chemical shifts and hence the conformations of V226^5.57^ at the intracellular side of TM5 and V314^6.59^ at the extracellular side of TM6 or alternatively those of V172^4.56^ close to the ligand head group fully span the space of the first two PCA components and therefore capture most on the receptor behavior. This high predictive power of the combined ^1^H-^15^N shifts of V172^4.56^ is very satisfying since the chemical nature of the ligand must be the cause of the structural variation.

**Fig. 5:**
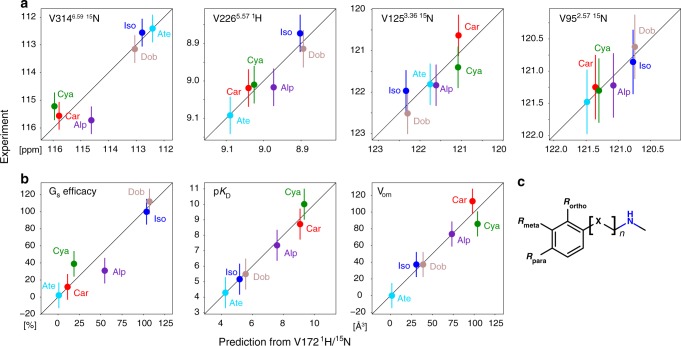
Prediction of receptor observables from V172^4.56^-^1^H/^15^N chemical shifts using PCA. Predictions were based on the first two principal components. **a** Predicted vs. observed chemical shifts V314^6.59^-^15^N, V226^5.57^-^1^H, V125^3.36^-^15^N, V95^2.57^-^15^N. **b** Predicted vs. observed G_s_ efficacy, ligand affinity p*K*_D_ and volume of ortho, meta ligand head group substitutions (*V*_om_). Error bars in **a** and **b** are estimates for the agreement between measured and predicted chemical shifts (^1^H: 0.05 ppm, ^15^N: 0.5 ppm) and ligand parameters (G_s_ efficacy: 15%, p*K*_D_: 1.0, *V*_om_: 15 Å^3^), which were based on the overall quality of the PCA fit. **c** Substitution pattern of the aromatic ligand head group. Source data are provided as a Source Data file.

Evidently only two linearly independent observations are sufficient to predict all other observations within 90% accuracy. As example we have chosen the ^1^H-^15^N chemical shifts of V172^4.56^ and calculated the respective matrix transformations. These chemical shifts then predict all other observed chemical shifts within 0.05 ppm for ^1^H and 0.3 ppm for ^15^N (Fig. 5a), as well as G_s_ efficacy, p*K*_D_ and *V*_om_ within 13%, 0.3 p*K* unit, and 10 Å^3^, respectively (Fig. 5b). Predictions of similar quality are obtained when using G_s_ efficacy and p*K*_D_, or V314^6.59^-^15^N/V226^5.57^-^1^H as input variables.

### The *C*_p_→*C*_a_ transition involves TM6 pivoting

The binding of G protein or G protein mimics strongly increases the affinity of agonist ligands to GPCRs^13^, amounting to a p*IC*_50_ shift from 4.4 to 6.3 for the YY-β_1_AR•isoprenaline complex^20^. Here we show that the unusual behavior of the V314^6.59^ backbone resonances provides insights into the driving forces of this transmembrane allosteric coupling and a simple mechanical explanation.

**Fig. 6:**
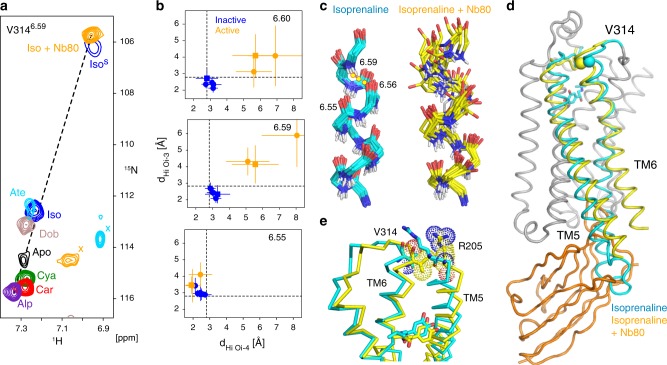
Ligand-induced chemical shift changes at the extracellular end of TM6. **a** Selected region from ^1^H-^15^N TROSYs showing the variation of the V314^6.59 1^H-^15^N resonances in the apo form and in orthosteric binary and ternary isoprenaline•Nb80 complexes of YY-β_1_AR at 21 T and 304 K. Due to limited stability, the apo form of YY-β_1_AR was recorded at 294 K. Spectra are color-coded and marked according to the ligand. Due to exchange broadening, the resonance marked by ‘Iso^s^’ was only observed at 14 T. It corresponds to the second, minor conformation of the YY-β_1_AR•isoprenaline complex and coincides with the resonance of V314^6.59^ resonance in the ternary isoprenaline•Nb80 complex (orange). Unrelated resonances are marked by ‘x’. A dashed line visualizes the approximate linear correlation between ^1^H and ^15^N chemical shifts of V314^6.59^ in various receptor states. **b** Hydrogen bond distances *d*_HiOi-4_ and *d*_HiOi-3_ for residues 6.54, 6.59 and 6.60 in β_2_AR (square) and β_1_AR (circle) from Phenix ensemble calculations for binary orthosteric ligand complexes (2Y03, 2RH1, 2VT4 chain A and B; blue) and ternary agonist complexes (4LDL, 6H7J; orange). Source data are provided as a Source Data file. **c** Ensemble backbone structures ranging from residue 6.50–6.59 derived by the Phenix ensemble calculations for β_1_AR•isoprenaline (2Y03, cyan) and β_1_AR•isoprenaline•Nb80 (6H7J, yellow). The 3_10_ hydrogen bond H^6.59^•••O^6.56^ in the agonist-bound structure is shown as a dotted line. **d** Evidence for the TM6 pivoting in response to nanobody binding in the crystal structures of β_1_AR. Structures of the binary complex with the agonist isoprenaline (2Y03) and of the ternary complex with agonist isoprenaline and Nb80 (6H7J) were aligned on the central region of the unaffected helices TM1-4,7 (shown in gray for 2Y03). TM5 and TM6 are depicted in cyan for the binary complex and in yellow for the ternary complex. The V314 N atoms are depicted as spheres, nanobody Nb80 as an orange ribbon. **e** Ligand-binding pocket of the binary and ternary complex β_1_AR structures as shown in **d**. Residues V314^6.59^, R205^5.37^ and ligands are depicted in stick representation. Van der Waals spheres are shown for V314^6.59^ and R205^5.37^ in the ternary complex to visualize their contact.

Strikingly, the addition of Nb80 to the YY-β_1_AR•isoprenaline complex further shifts the main V314^6.59 1^H/^15^N resonance by ~0.3/7 ppm in the upfield direction from its already unusual position, such that the ^1^H/^15^N V314^6.59^ resonances of all YY-β_1_AR complexes and of its apo form fall approximately on a single line (Fig. 6a). As mentioned before, the minor V314^6.59 1^H/^15^N resonance of the binary YY-β_1_AR•isoprenaline complex is at the same position as that of the ternary complex, proving that this ‘active’ conformation is already populated to about 20% in the binary complex. The remarkable linear correlation over 10 ppm for ^15^N and 0.4 ppm for ^1^H chemical shifts for all YY-β_1_AR complexes indicates that the average conformations of V314^6.59^ follow a continuous, smooth path in response to all ligands including the G protein-mimicking nanobody.

The extreme ^15^N upfield shift of V314^6.59^ in the β_1_AR•isoprenaline•Nb80 complex must be caused by a considerable weakening of the H-bond from the N atom and a strong distortion of the backbone geometry of V314^6.59,36^. Indeed, a detailed analysis of available β_1_AR and β_2_AR structures by the phenix.ensemble_refinement module^37^ corroborates this observation. This program fits experimental structure factors by an ensemble of structural models to account for molecular disorder. Within the variations of the ensembles of all available binary complex structures of β_1_AR or β_2_AR with agonists or antagonists, TM6 residues 6.59 (V314 in β_1_AR, V297 in β_2_AR) and 6.60 form 3_10_-helical H_i_•••O_i-3_ H-bonds. This is exemplarily shown in Fig. 6b, c for the β_1_AR complexes with cyanopindolol (PDB 2VT4) and isoprenaline (PDB 2Y03) and the β_2_AR complex with carazolol (PDB 2RH1). In contrast, in the ternary complexes of agonist-bound β_1_AR and β_2_AR with the G protein-mimicking nanobodies Nb80 (PDB 6H7J) and Nb6B9 (PDB 4LDL), respectively, the H^6.59^•••O^6.56^ and H^6.60^•••O^6.57^ H-bonds are clearly broken (*d*_HO_ > 3.0 Å, Fig. 6b, c), in complete agreement with the strong upfield shift of the V314^6.59 15^N and ^1^H resonances^36^. Thus apparently, the conformational change to the active conformation in this region exerts forces onto the extracellular end of TM6.

The cause for these forces becomes apparent when the agonist isoprenaline-bound β_1_AR structure (PDB 2Y03) is aligned onto the ternary complex of β_1_AR with isoprenaline and Nb80 (PDB 6H7J) on the central region of helices TM1-4,7 (Fig. 6d). Clearly visible is the large displacement of transmembrane helices 5 and 6 (TM5,6) by up to 14 Å outward from the transmembrane 7-helix bundle, which is the hallmark of G protein^38^ or G protein-mimicking nanobody binding^13,39^. The TM6 motion extends to the extracellular end of TM6. This part moves in the opposite direction of the intracellular part towards the orthosteric ligand-binding pocket, corresponding to a pivoting motion of TM6 around its center close to the conserved P305^6.50^. A concomitant sideways motion occurs on the extracellular end of TM5 and the ligand itself inserts more deeply into the binding pocket with its catechol group forming polar interactions^39,40^ with N310^6.55^ and S211^5.42^ (Fig. 6e). As a consequence of the TM6 pivoting, residue V314^6.59^ moves by 2.4 Å towards the ligand. Apparently, a steric contact between residues V314^6.59^ and R205^5.36^ transmits force between the extracellular ends TM6 and TM5 (Fig. 6e), which in turn strains the backbone at V314^6.59^ and causes its extreme ^15^N chemical shift.

An identical TM6 motion and the formation of very similar contacts are observed in the β_2_AR ternary complex with the agonist HBI and nanobody Nb6B9 (PDB 4LDL) relative to the carazolol-bound β_2_AR structure (PDB 2RH1). Furthermore, also the orientations of the extra and intracellular parts of TM5 and TM6 in the complex of β_2_AR with the agonist BI167107 and G protein (PDB 3SN6) are very similar to the β_2_AR•HBI•Nb6B9 complex, albeit low electron density at the extracellular side prevents a precise definition of individual atoms^38^. Therefore, the TM6 pivoting motion appears conserved at least for the beta-adrenergic receptors upon nanobody or G protein binding.

The TM6 pivoting provides a simple mechanical rationale for the proposed ligand pocket compression by effector proteins and the increase in agonist affinity by tighter contacts in the ligand pocket. The pivoting mechanism may also explain the antagonistic pharmacology of high-affinity ligands with large hydrophobic ortho and meta substitutions. The latter impede the inward motion of the extracellular part of TM6 and thereby the opening of the intracellular effector site for G protein (mimic) binding.

### The Y^5.58^-Y^7.53^ bridge stabilizes active conformation of TM6

**Fig. 7:**
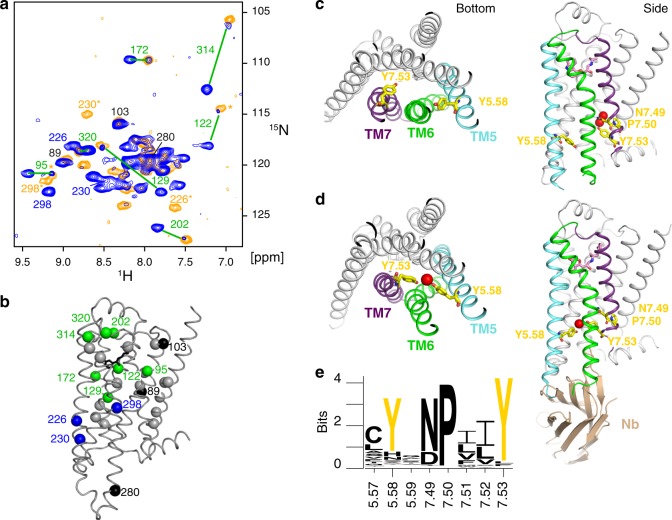
Role of conserved tyrosines Y^5.58^ and Y^7.53^ in the activation of β_1_AR. **a** ^1^H-^15^N TROSY spectra of ^15^N-valine-labeled YY-β_1_AR in the binary complex with isoprenaline (blue) and in the ternary complex with isoprenaline and Nb80 (orange) at 21 T. Resonances are marked with assignment information. Green lines connect resonances corresponding to the major (preactive) and minor (active) conformation in the binary isoprenaline complex where the resonances of the minor conformation coincide with the ternary complex. Assignments marked by asterisks are tentative for the ternary complex. The minor V314 resonance of the YY-β_1_AR•isoprenaline complex was only observed in a spectrum recorded at 14 T (Fig. 6). **b** Structure of β_1_AR in complex with isoprenaline (2Y03). Valines are shown as spheres color-coded according to the spectral observation in **a**. Green: minor conformation observed in the binary YY-β_1_AR•isoprenaline complex, which coincides with the ternary YY-β_1_AR•isoprenaline•Nb80 complex. Blue: no second conformation observed in the binary complex. Black: no change between binary and ternary complex. Gray: no assignment available in the ternary complex. **c**, **d** Bottom and side view of tyrosines Y^5.58^ and Y^7.53^ in the structures of β_2_AR in a binary antagonist complex (2RH1, **c**) and a ternary agonist•Nb6B9 complex (4LDE, **d**). Y^5.58^, N^7.49^, P^5.50^ and Y^7.53^ are depicted as sticks (CPK-yellow). Water molecules involved in H-bonds with Y^5.58^ and Y^7.53^ are shown as red spheres. Helices TM5, TM6, TM7 and Nb6B9 are indicated by different colors. **e** Conservation of Y^5.58^ and the NPxxY^7.53^ motif within all 1554 class A GPCR entries in the GPCRdb^59^. Source data are provided as a Source Data file.

As indicated above, the binary YY-β_1_AR•isoprenaline complex shows a second set of weak resonances for certain residues that coincide with those of the respective residues in the *C*_a_ conformation of the ternary YY-β_1_AR•isoprenaline•Nb80 complex. This is the case for V172^4.56^, V202^ECL2^, V314^6.59^, V95^2.57^, V122^3.33^, V129^3.40^ and V320^ECL3^ (Fig. 7a), of which V95^2.57^ and V122^3.33^ are currently only tentatively assigned in the ternary complex. These residues are located within the extracellular half of β_1_AR around the ligand-binding pocket (Fig. 7b). Thus at least this part of the YY-β_1_AR•isoprenaline complex is in a slow equilibrium on the chemical shift timescale between the preactive *C*_p_ and the active *C*_a_ conformation even in the absence of the Nb80 effector protein. From the observed frequency separation and the intensity ratios, the exchange between the two conformations must be slower than ~5 ms and about 20% of the extracellular half of the receptor is in conformation *C*_a_. In contrast, no second resonances corresponding to the ternary complex were observed for residues V226^5.57^, V230^5.61^ and V298^6.43^ above the noise threshold (10% of *C*_p_ intensity, Fig. 7a). The latter are located within the intracellular half of the receptor close to the effector binding site. The absence of resonances at the respective *C*_a_ positions for these residues may have several causes: (i) additional exchange broadening of the binary complex in this region, (ii) these residues populate only the *C*_p_ conformation in the absence of an effector protein, (iii) they occupy a further conformation, which has not yet been identified. In all cases, this finding indicates that the intra- and extracellular sites are not completely coupled. Of note, resonances of V89^2.52^, V103^2.65^ in TM2 and V280^6.25^ at the intracellular end of TM6 coincide for the YY-β_1_AR•isoprenaline and YY-β_1_AR•isoprenaline•Nb80 complex. Therefore the conformations at these locations are similar for *C*_p_ and *C*_a_.

In contrast to the YY-β_1_AR•isoprenaline complex, the TS-β_1_AR•isoprenaline complex does not show a second set of resonances above the noise level (12% of the C_p_ intensities). This coincides with the fact that only YY-β_1_AR•isoprenaline, but not TS-β_1_AR•isoprenaline form the active complex with G protein or Nb80^20^. Therefore tyrosines Y227^5.58^ and Y343^7.53^ are clearly required for the transition to the active conformation of β_1_AR and the effector protein binding apparently occurs to this preformed conformation of the binary YY-β_1_AR•isoprenaline complex. Both Y227^5.58^ and Y343^7.53^, which is part of the NPxxY motif, are highly (>76%) conserved in all class A GPCRs (Fig. 7e). Mutations of these tyrosines lead to decreased sequestration of β_2_AR^41^, a decreased lifetime of the rhodopsin meta II intermediate^42^, and a stabilization of the inactive state in α_1B_AR and β_2_AR^43^.

As all solved binary and ternary β_1_AR complex structures either carry mutations in Y227^5.58^ and Y343^7.53^ or are of relatively low resolution, the best structural insight for these tyrosines and the associated NPxxY motif is obtained from the higher-resolution β_2_AR complexes. In the complex of β_2_AR with the antagonist carazolol (PDB 2RH1), the side chain of Y^7.53^ orients towards the side chain of N^7.49^ of the NPxxY motif forming a water-mediated H-bond network, whereas the side chain of Y^5.58^ points towards the intracellular opening of the transmembrane helix bundle (Fig. 7c). In contrast, in the ternary agonist•β_2_AR•Nb6B9 (PDB 4LDE) and agonist•β_2_AR•G protein (PDB 3SN6) complexes the side chains of Y^5.58^ and Y^7.53^ face each other and connect via a water molecule, which is visible in the higher-resolution 4LDE structure (Fig. 7d). This water-mediated bridge between the two tyrosines subtends TM6 and apparently stabilizes its swung-out active conformation.

As the active conformation *C*_a_ is only observed by NMR in the presence of the two tyrosines, we identify this conformation with the closed state of the Y^5.58^-Y^7.53^ bridge. This observation for residues in the extracellular half of the receptor also proves a direct coupling of the state of the tyrosine bridge to this part of the protein. The observed slow exchange (>5 ms) between *C*_p_ and *C*_a_ agrees well with the expected slow timescale required for the substantial rearrangement of the receptor during the closing of this bridge, which comprises the outward motion of TM6 and the rearrangement of the tyrosine side chains and their associated H-bond network. Our data show that the closing of the tyrosine bridge can already occur in the absence of an intracellular binding partner. However, the free energy stabilization by the bridge formation is not large enough to achieve full occupancy of the active conformation. The latter is only reached by an additional stabilization from the bound G protein or nanobody.

### Modulation of β_1_AR dynamics by ortho- and allosteric ligands

**Fig. 8:**
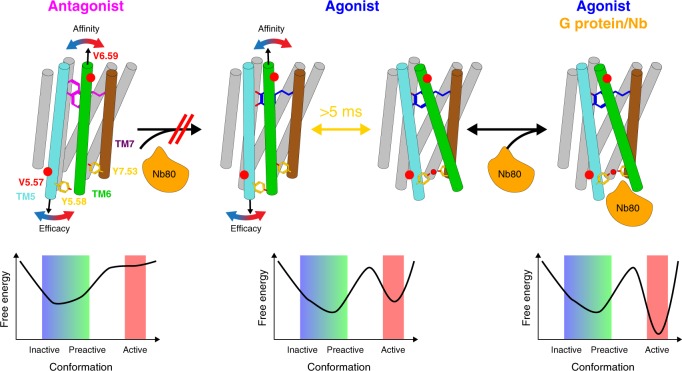
Overview of observed β_1_AR conformational dynamics in response to ligands. Top: overview of conformational averages in various ligand-bound forms: (i) the antagonist complex in fast equilibrium biased to an inactive conformation, (ii) the agonist complex in fast equilibrium biased towards the preactive conformation and in a further slow equilibrium (closing of YY-bridge) with the active conformation, (iii) the ternary agonist•effector protein complex in the fully active conformation. Arrows indicate readouts of conformational averages provided by chemical shifts that correlate to ligand efficacy (V^5.57^) and affinity (V^6.59^). YY-bridge residues Y^5.58^ and Y^7.53^ are shown in yellow. Bottom: free energy landscape corresponding to the various ligand-bound forms.

Figure 8 summarizes the insights from the present study into the conformational dynamics of the β_1_AR as a function of ligand binding. On a timescale faster than about 100 μs, binary receptor complexes with orthosteric ligands undergo conformational exchange between various subconformations with their populations modulated by properties of the ligand such as efficacy for G protein activation or affinity. As this exchange is faster than the chemical shift variation between subconformations, a single NMR resonance is observed, which shifts according to the ligand properties, e.g. V314^6.59^ (affinity) and V226^5.57^ (efficacy). These subconformations involve motions in TM5 and TM6. A principal component analysis of the averaged chemical shifts from many locations in the receptor and of the ligand properties shows that the entire receptor behavior within this fast equilibrium can be described to high accuracy by a simple two-dimensional continuum of parameters. On a timescale slower than about 5 ms, a transition occurs for the preactive agonist-bound receptor from its fast equilibrium conformational average to a new conformation populated at about 20%. This conformation is highly similar to the active conformation observed in the ternary agonist•Nb80 complex. The transition involves the closure of the water-mediated bridge between Y^5.58^ and Y^7.53^, which stabilizes TM6 in its swung-out position at the intracellular side. The active conformation gets fully populated by the intracellular binding of G protein or a G protein-mimicking nanobody, which further stabilize the swung-out position of TM6. The TM6 motion extends to the extracellular side corresponding to a pivoting around its center, which results in the compression of the ligand pocket in the active conformation and a concomitant increase in agonist affinity. The pivoting is impeded for antagonist ligands with large hydrophobic head group substitutions providing a rationale for reduced effector binding.

## Discussion

We have obtained an in-depth description of the backbone dynamics, conformational landscape and activation mechanism of the β_1_AR as a paradigm for class A GPCRs using ^15^N NMR relaxation rates and ^1^H-^15^N chemical shifts. We can distinguish several dynamic, conformational equilibria, which are orchestrated by the binding of orthosteric ligands and an intracellular G protein-mimicking nanobody. These comprise an inactive–preactive conformational equilibrium exchanging on a timescale faster than about 100 μs and a second transition to an active conformation, which is slower than about 5 ms.

The ligand-induced chemical shift variations within these equilibria indicate a pivoting motion of TM6 that extends from the intracellular to the extracellular side. Rigid-body motions of TM6 have been postulated for rhodopsin^44^ and β_2_AR^45^ before the availability of detailed crystal structures. However, the motion of the extracellular part of TM6 was predicted towards TM7 and not TM5^45^. More recently, the observed formation of a lid-like structure by F193^ECL2^ and Y308^7.35^ over the orthosteric ligand-binding site in β_2_AR^46^ as well as a general compression of the ligand-binding pocket in β_1_AR^34,39^ upon binding of G protein or nanobodies have been used to explain the hundredfold increase in agonist affinity^13,20^ in the presence of effector proteins. Here we provide direct evidence that the intracellular G protein (mimic) binding pushes the extracellular part of TM6 towards TM5 and thereby compresses the ligand-binding pocket. This motion is sterically hindered by ligands with large hydrophobic ortho and meta substitutions at the benzene ring explaining their antagonist pharmacology.

TM6 pivoting with its relation to ligand affinity captures only part of the receptor regulation, since they are not correlated with the efficacy for the G_s_ pathway. A combined PCA of all observed valine ^1^H-^15^N chemical shifts, ligand p*K*_D_s, efficacies and volume of ligand head group substitutions reveals that the total observed receptor behavior on the fast-timescale of the inactive–preactive equilibrium requires two linearly independent parameters for an accurate description. One of these parameters may be identified with ligand affinity and the correlated TM6 pivoting as detected by the V314^6.59^ chemical shifts, the second may be identified with ligand efficacy and the correlated V226^5.57^ chemical shift. The latter connection presumably involves mechanical pathways from the ligand to the receptor site via TM5.

The supra-millisecond transition time from the preactive to the active conformation agrees well with millisecond fluctuations observed in agonist-bound β_2_AR by single molecule fluorescence^47^ and with the 1–2 ms time required for the transition of light-activated rhodopsin to the active metarhodopsin II state^48^. It also is compatible with times of tens of milliseconds observed for active-state transitions by in vivo FRET experiments on the β_1_AR^49^ and other non-rhodopsin GPCRs^48^ considering that the latter experiments are limited in time resolution by the mixing and diffusion of ligands^49^.

The comparison between the YY-β_1_AR and TS-β_1_AR mutants revealed that the formation of the water-mediated Y^5.58^-Y^7.53^ bridge is required for the transition to the active conformation and G protein (mimic) binding. Its formation induces allosteric conformational changes around the extracellular binding pocket. The bridge forms in the agonist-bound receptor already to about 20% in the absence of an intracellular effector protein. The overall active conformation is then further stabilized via conformational selection when the G protein-mimicking nanobody binds. Several residues at the intracellular side do not show an active conformation above the noise threshold in the absence of nanobody. This may indicate that intra- and extracellular sides are not very tightly coupled in agreement with suggestions that GPCRs function as a network of loosely coupled microswitches^22,28,50^. Further NMR data in the intracellular region are required to clarify and quantify this issue.

The presented conclusions are based on an extensive analysis of receptor dynamics determined from NMR ^15^N relaxation data and a comparison of ^15^N chemical shifts to existing crystal structures. The chemical shifts of backbone ^15^N nuclei are well understood and can be directly linked to defined backbone conformations. Such correlations are much less stringent for side chain nuclei. The variations in ^15^N shifts show that minute motions in the crystal structures, which may otherwise be considered as ‘structural noise’, are indeed highly significant for function. Thus the combination of NMR and crystallographic information gives precise insights into the driving forces of biological function.

## Methods

### Protein expression and purification

Expression in baculovirus-infected Sf9 cells (Oxford Expression Technologies) and purification of the ^15^N-valine-labeled turkey TS-β_1_AR and YY-β_1_AR constructs were carried out as described previously^20^. Uniformly, ^15^N(~90%), ^2^H(~60%)-labeled TS-β_1_AR was obtained by supplementation of ^15^N, ^2^H-labeled yeastolate and ^15^N_2_-glutamine to custom-made serum-free medium (SF4, BioConcept) devoid of amino acids and yeast extract medium as described^32^. For all constructs, binding of ligands and exchange between ligands was carried out as described^20^. The plasmid for Nb80 was a generous gift by Jan Steyaert and the Nb80 protein was purified according to the described procedure^13^.

### NMR experiments and data analysis

NMR samples were prepared with typical receptor concentrations of 100–150 μM in 20 mM TRIS HCl, 100 mM NaCl, ~1% DM, 0.02% NaN_3_, 5% D_2_O_,_ pH 7.5 and 1 mM ligand (except apo form) solution in a 270 μl Shigemi tube, following concentration of ~15 μM receptor solubilized in 0.1% DM with a 50 kDa molecular weight cut-off centrifugal filter (Amicon). 2 mM sodium ascorbate was added to isoprenaline and dobutamine complex samples to prevent oxidation of the ligand.

All NMR experiments were performed on Bruker AVANCE 14.1 T (600 MHz ^1^H frequency), 18.8 T (800 MHz), or 21.2 T (900 MHz) spectrometers equipped with TCI cryoprobes at a temperature of 304 K with the exception of the apo YY-β_1_AR ^1^H-^15^N TROSY, which was recorded at 294 K due to limited stability. ^1^H-^15^N TROSY experiments were recorded as 120 or 80 (^15^N) × 1024 (^1^H) complex points and acquisition times of 24 ms or 16 ms (^15^N) and 42 ms (^1^H). For optimal sensitivity, the ^1^H-^15^N transfer time was reduced to 3.0 ms. The experimental times using a 1 s interscan delay were adjusted to reach signal to noise ratios of ~10 for the analyzed peaks, corresponding to ~24 h for a typical 100 μM receptor sample.

^15^N *R*_1_ and *R*_2_ relaxation rates were determined using standard HSQC-based ^15^N relaxation experiments^35,51^ with relaxation delays of 20, 2004 ms (20, 3004 ms) for *R*_1_ and 2, 6, 12 ms (2, 6, 10 ms) for *R*_2_ at 14.1 T (21.2 T). ^15^N-^1^H dipolar-coupling/^15^N CSA cross-correlation rates η were determined from a quantitative comparison of in-phase and anti-phase ^15^N magnetization^52^ using a cross relaxation delay of 12 ms.

All NMR spectra were processed with NMRPipe^53^ and evaluated with SPARKY^54^ or PIPP^55^. Resonance amplitudes of NMR relaxation spectra were extracted using the program nlinLS contained in NMRPipe^53^. ^15^N *R*_1_ and *R*_2_ rates and their error estimates were obtained by Monte Carlo fitting of the experimental amplitudes using Matlab (MATLAB_R2016B, MathWorks, Inc.).

^15^N *R*_2_ rates were also determined by time-domain line shape fitting of ^1^H-^15^N TROSY spectra using the nlinLS program contained in NMRPipe^53^. For this, two-dimensional ^1^H-^15^N resonances were created as exponentially damped sinusoids, apodized, one-time zero-filled and Fourier transformed with the same parameters as the experimental data. Amplitudes, frequencies and decay constants were then varied to obtain the best least squares fit to the experimental spectra. The quality of the fit is documented for specific examples in Supplementary Fig. 4. A Monte Carlo error analysis of the fit parameters was carried out on synthetic spectra created with random noise of the same root mean square as the experimental data. Reported errors on the fitted *R*_2_ rates are standard deviations of fitted *R*_2_ values of 20 such Monte Carlo simulations.

### Multi-angle light scattering and viscosity measurements

SEC-MALS measurements on TS-β_1_AR in DM micelles were carried out at 299 K using a GE Healthcare Superdex 200 Increase 10/300 size-exclusion column on an Agilent 1260 HPLC with a column buffer of 20 mM TRIS HCl, 100 mM NaCl, pH 7.5 containing 0.76% DM to match the NMR sample conditions. Elution was monitored by an absorbance detector (280 nm), a Wyatt Heleos II 8+ multi-angle light-scattering detector and a Wyatt Optilab rEX differential refractive index detector. Inter-detector delay volumes, band broadening corrections and light-scattering detector normalization were calibrated using 2 mg/ml BSA solution (ThermoPierce) and standard protocols in ASTRA 6. The refractive index increment (d*n*/d*c*) of DM in column buffer was calculated as 0.141 ml/g from measurements of a series of samples with concentrations between 0.5 and 10 mg/ml, directly injected into the differential refractive index detector. Weight-averaged molar masses (Mw) for the protein-detergent complex (101 ± 2 kDa), and for the protein (36 ± 2 kDa) and detergent components (65 ± 3 kDa) of the complex were calculated using the protein conjugate method in the ASTRA 6 software (Wyatt Technology).

The viscosity of the receptor micelle suspension was estimated from a viscosity measurement of a 20 mM TRIS HCl, 100 mM NaCl, pH 7.5, 0.95% DM suspension using an Anton Paar AMVn rolling-ball viscometer yielding a value of 0.840 cP at 304 K.

### Theoretical relaxation rates

Theoretical ^15^N *R*_1_(N_z_), in-phase *R*_2_(N^+^)^35^, ^15^N-^1^H dipolar-coupling/^15^N CSA cross-correlation η^52^ and TROSY *R*_2_(N^+^H_β_)^56^ rates were calculated using an isotropic Lipari–Szabo spectral density and the following parameters: *r*_HN_ = 1.02 Å, *S*^2^ =0.85, ∆*σ*_N_ = 170 ppm, ∆*σ*_HN_ = 15 ppm, *θ* = 20°, where *θ* is the angle between the unique axes of the CSA and dipolar tensors. Isotropic rotational correlation times *τ*_c_ were determined independently from measured ^15^N *R*_1_(N_z_) rates and ^15^N-^1^H dipolar-coupling/^15^N CSA cross-correlation rates *η* by inversion of the respective theoretical expressions. Effects of dipolar interactions from nearby protons onto the anti-phase ^15^N transverse relaxation rates in ^1^H-^15^N TROSY experiments were taken into account by the addition of half the *R*_1,sel_(H_z_) rate with *r*_HH_ = 2.05 Å as an effective distance corresponding to the dipolar interaction with all protons adjacent to the amide proton in α-helical structures^56^. The theoretical equations used for the ^15^N relaxation rates are

1 $$R_1\left( {{\mathrm{N}}_z} \right) = \frac{{3 B_0^2{\mathrm{\Delta }}\sigma _{\mathrm{N}}^2\gamma _{\mathrm{N}}^2}}{{10}}{J\left( {\omega _{\mathrm{N}}} \right)} + \frac{{\mu _0^2h^2\gamma _{\mathrm{H}}^2\gamma _{\mathrm{N}}^2}}{{160\pi ^2r_{{\mathrm{NH}}}^6}}\left[ {6J\left( {\omega _{\mathrm{H}} + \omega _{\mathrm{N}}} \right) + J\left( { - \omega _{\mathrm{H}} + \omega _{\mathrm{N}}} \right) + 3J\left( {\omega _{\mathrm{N}}} \right)} \right]$$

2 $$R_2\left( {{\mathrm{N}}^ + } \right) 	= \frac{{B_0^2{\mathrm{\Delta }}\sigma _{\mathrm{N}}^2\gamma _{\mathrm{N}}^2}}{{20}}\left[ {4J\left( 0 \right) + 3J\left( {\omega _{\mathrm{N}}} \right)} \right] \;\\ 	+ \frac{{\mu _0^2h^2\gamma _{\mathrm{H}}^2\gamma _{\mathrm{N}}^2}}{{320\pi ^2r_{{\mathrm{NH}}}^6}}\left[ {4J\left( 0 \right) + 6J\left( {\omega _{\mathrm{H}}} \right) + 3J\left( {\omega _{\mathrm{N}}} \right) + J\left( { - \omega _{\mathrm{H}} + \omega _{\mathrm{N}}} \right) + 6J\left( {\omega _{\mathrm{H}} + \omega _{\mathrm{N}}} \right)} \right]$$

3 $$\eta = \frac{{B_0\Delta \sigma _{\mathrm{N}}\mu _0h\gamma _{\mathrm{H}}\gamma _{\mathrm{N}}^2}}{{40\pi r_{{\mathrm{NH}}}^3}}P_2(\cos \theta )[4J\left( 0 \right) + 3J\left( {\omega _{\mathrm{N}}} \right)]$$

4 $$R_{1 \rm{,sel}}\left( {{\mathrm{H}}_{\mathrm{z}}} \right) = \frac{{\mu _0^2h^2\gamma _{\mathrm{H}}^4}}{{160\pi ^2r_{{\mathrm{HH}}^a}^6}}[J\left( 0 \right) + 3\left( {\omega _{\mathrm{H}}} \right) + 6J\left( {2\omega _{\mathrm{H}}} \right)]$$

5 $${{R}}_2\left( {{\mathrm{N}}^ + {\mathrm{H}}_{\mathrm{z}}} \right) = 	\, \frac{{B_0^2\Delta \sigma _{\mathrm{N}}^2\gamma _{\mathrm{N}}^2}}{{20}}[4J(0) + 3J\left( {\omega _{\mathrm{N}}} \right)] + \frac{{\mu _0^2h^2\gamma _{\mathrm{H}}^2\gamma _{\mathrm{N}}^2}}{{320\pi ^2r_{{\mathrm{NH}}}^6}}[4J\left( 0 \right) + 3J\left( {\omega _{\mathrm{N}}} \right) \\ 	+ J\left( { - \omega _{\mathrm{H}} + \omega _{\mathrm{N}}} \right) + 6J\left( {\omega _{\mathrm{H}} + \omega _{\mathrm{N}}} \right)]$$

6 $${{R}}_2\big( {{\mathrm{N}}^ + {\mathrm{H}}_\beta ,{\mathrm{TROSY}}} \big) = \frac{{{{R}}_2\left( {{\mathrm{N}}^ + } \right) + {{R}}_2\left( {{\mathrm{N}}^ + {\mathrm{H}}_{\mathrm{z}}} \right) + {{R}}_{1 \rm{,sel}}\left( {{\mathrm{H}}_{\mathrm{z}}} \right)}}{2} - \eta .$$

An independent estimate of the rotational correlation time *τ*_c_ was obtained from the 101-kDa molecular weight of the TS-β_1_AR DM micelle complex determined by SEC-MALS. Using literature values^57^ for the partial specific volumes of protein (average value 0.735 ml/g, 0.36 mass fraction) and DM (0.815 ml/g, 0.64 mass fraction), the specific volume for the complex can be calculated as 0.786 ml/g. The non-hydrated radius *r*_NH_ of an assumed spherical detergent micelle is then 31.6 Å. Assuming a hydration layer thickness *r*_W_ of 3.2 Å, the isotropic rotational correlation time *τ*_c_ of the hydrated receptor detergent complex amounts to 35.2 ns using the Stokes–Einstein relation $\tau _{\mathrm{c}} = {\textstyle{{4\pi \eta _{\mathrm{S}}r_{\mathrm{H}}^3} \over {3kT}}}$ with *r*_H_ = *r*_NH_ + *r*_W_ and $\eta _{\mathrm{S}}$ being the viscosity of the receptor micelle suspension.

### Principal component analysis

The principal component analysis of chemical shift variations and ligand properties was carried out using NumPy.

### Phenix ensemble calculations

Ensembles refinements of various β_1_AR and β_2_AR crystal structures were calculated using the phenix_ensemble_refinement module^37^ of the Phenix software (version 1.14-3260). Average *d*_HiOi-4_ and *d*_HiOi-3_ distances over all ensemble entries and their standard deviations were determined separately for each chain. Molecular representations were generated using the PyMOL Molecular Graphics System (Schrodinger, LLC).

### Reporting summary

Further information on research design is available in the Nature Research Reporting Summary linked to this article.

## Supplementary information

Supplementary Information

Description of Additional Supplementary Files

Supplementary Data 1-3

Reporting Summary

## Data Availability

Data supporting the findings of this manuscript are available from the corresponding author upon reasonable request. A reporting summary for this Article is available as a Supplementary Information file. The source data underlying Figs. 4a–c, 5, 6b and 7e are provided as a Source Data file.

## Source data

Source Data
