# Supplementary material for: A high-resolution description of β1-adrenergic receptor functional dynamics and allosteric coupling from backbone NMR
Source: Nat Commun. 2020 May 5;11:2216. doi: 10.1038/s41467-020-15864-y (PMC7200737; doi:10.1038/s41467-020-15864-y)
Supplement: Supplementary file 1 — Supplementary Information [file 41467_2020_15864_MOESM1_ESM.pdf]

A high-resolution description of β_1-adrenergic receptor functional dynamics and allosteric coupling from backbone NMR

Anne Grahl[1], Layara Akemi Abiko[1], Shin Isogai[1], Timothy Sharpe[2], Stephan Grzesiek[1,*]

[1] Focal Area Structural Biology and Biophysics, Biozentrum, University of Basel, CH-4056 Basel, Switzerland

[2] Biophysics Core Facility, Biozentrum, University of Basel, CH-4056 Basel, Switzerland

Supplementary Data

*Address correspondence to:
Stephan Grzesiek
Focal Area Structural Biology and Biophysics, Biozentrum
University of Basel, CH-4056 Basel, Switzerland
Phone: ++41 61 267 2100
FAX: ++41 61 267 2109
Email: Stephan.Grzesiek@unibas.ch

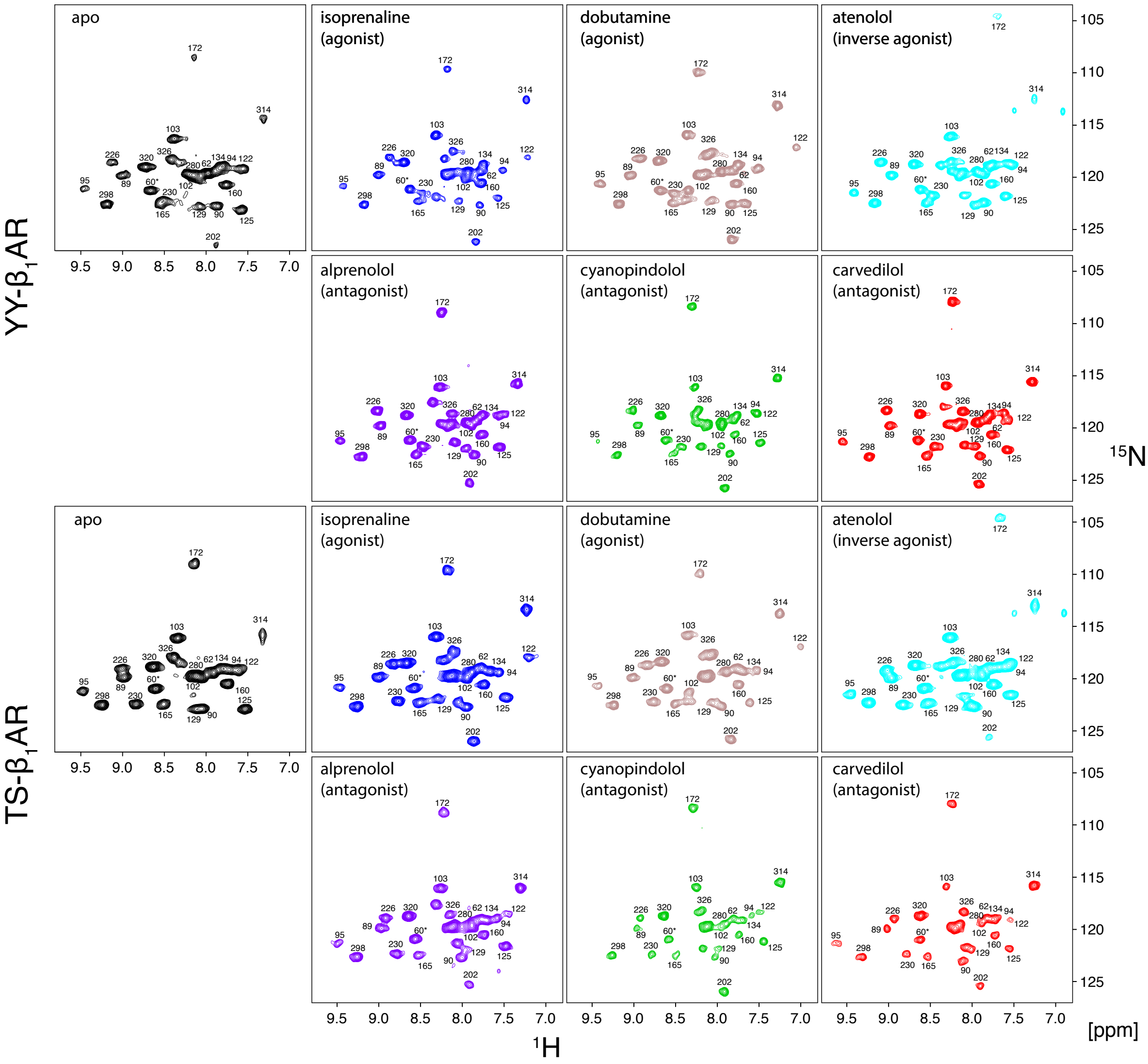

Supplementary Figure 1. ^{1}H-^{15}N TROSY spectra of ^{15}N-valine-labeled β$_1$AR. YY-β$_1$AR (top) and TS-β$_1$AR (bottom) in decyl-maltoside micelles in apo form and binary complexes. Resonances are marked with assignment information (asterisks for tentative).

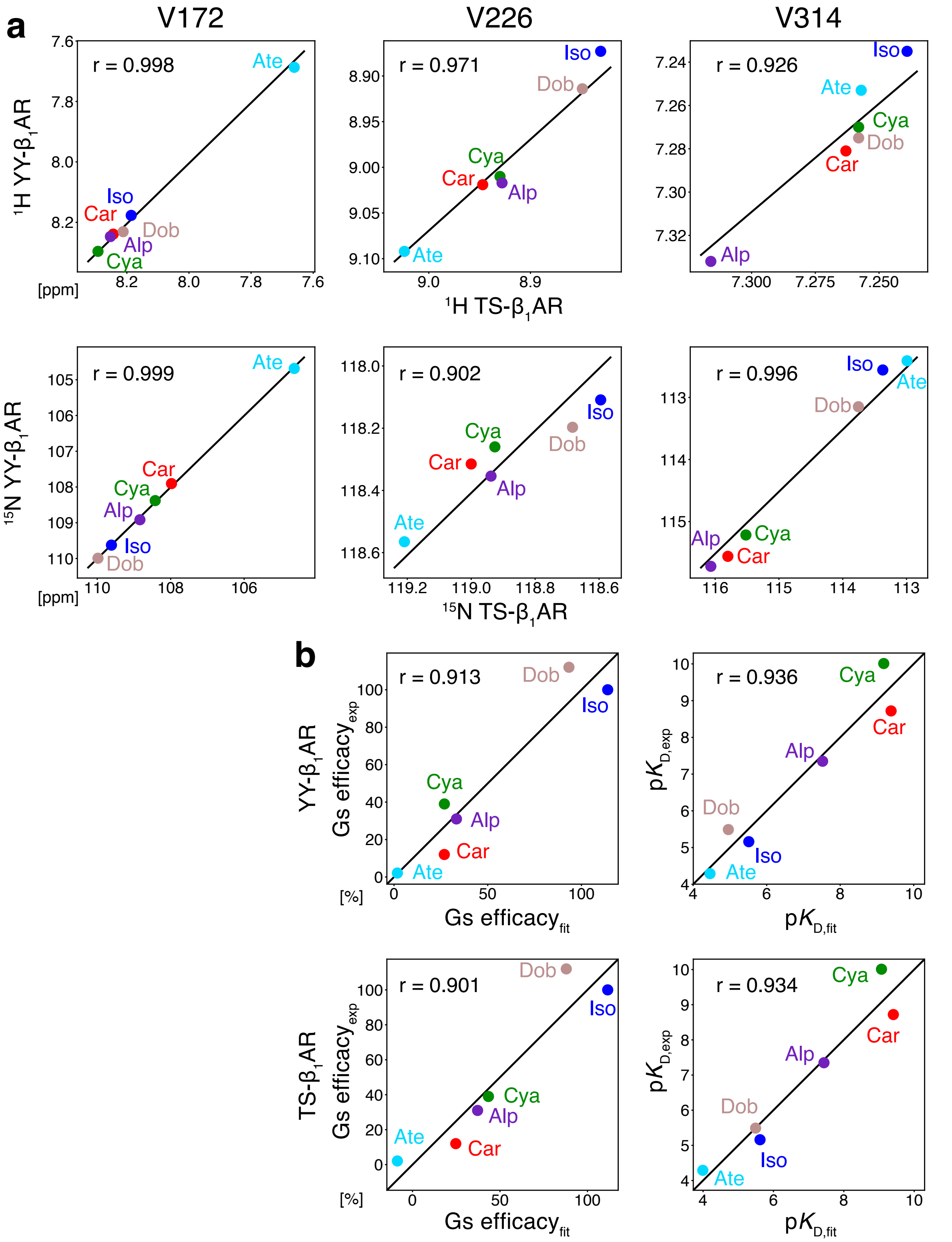

Supplementary Figure 2. Quantitative comparison of V172$^{4.56}$, V226$^{5.57}$, V314$^{6.59}$ ^{1}H-^{15}N chemical shifts in orthosteric ligand complexes of YY-β$_1$AR and TS-β$_1$AR. (a) Comparison of ^{1}H and ^{15}N shifts. (b) Correlation of experimental values for G$_s$ efficacy (G$_s$ efficacy$_{exp}$, left) and pK_D (p$K_{D,exp}$, right) to fitted linear combinations of chemical shifts (a·δ^{1}H + b·δ^{15}N + c) of V226$^{5.57}$ (G$_s$ efficacy$_{fit}$, left) and V314$^{6.59}$ (p$K_{D,fit}$, right) for YY-β$_1$AR (top row) and TS-β$_1$AR (bottom row). Numerical data are provided in Supplementary Data 2.

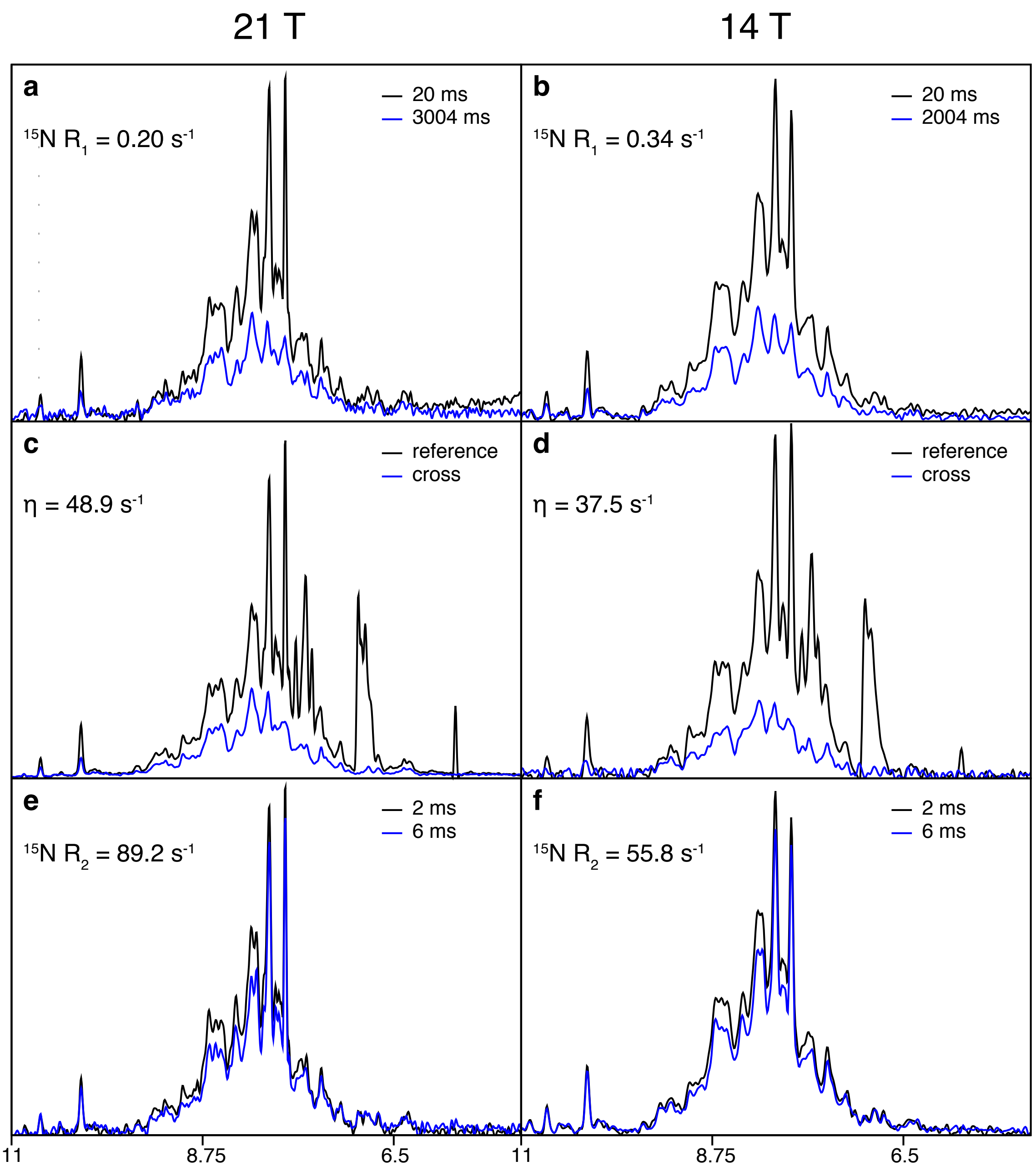

Supplementary Figure 3. Global backbone dynamics of β$_1$AR determined from 1D ^{1}H-detected ^{15}N relaxation experiments. Experiments were carried out on ^{2}H (~60%)/^{15}N-labeled, alprenolol-bound TS-β$_1$AR in DM micelles at 21 T (a,c,e) and 14 T (b,d,f). Relaxation rates were determined from intensity ratios of attenuated (blue) vs. reference spectra (black). Determined relaxation rates as well as relaxation delays for R_1 and R_2 experiments are indicated. (a,b) ^{15}N longitudinal (R$_1$) relaxation experiments. (c,d) ^{15}N-^{1}H dipolar coupling/^{15}N CSA cross-correlation experiments. The transfer time was 12 ms. (e,f) ^{15}N transverse (R$_2$) relaxation experiments.

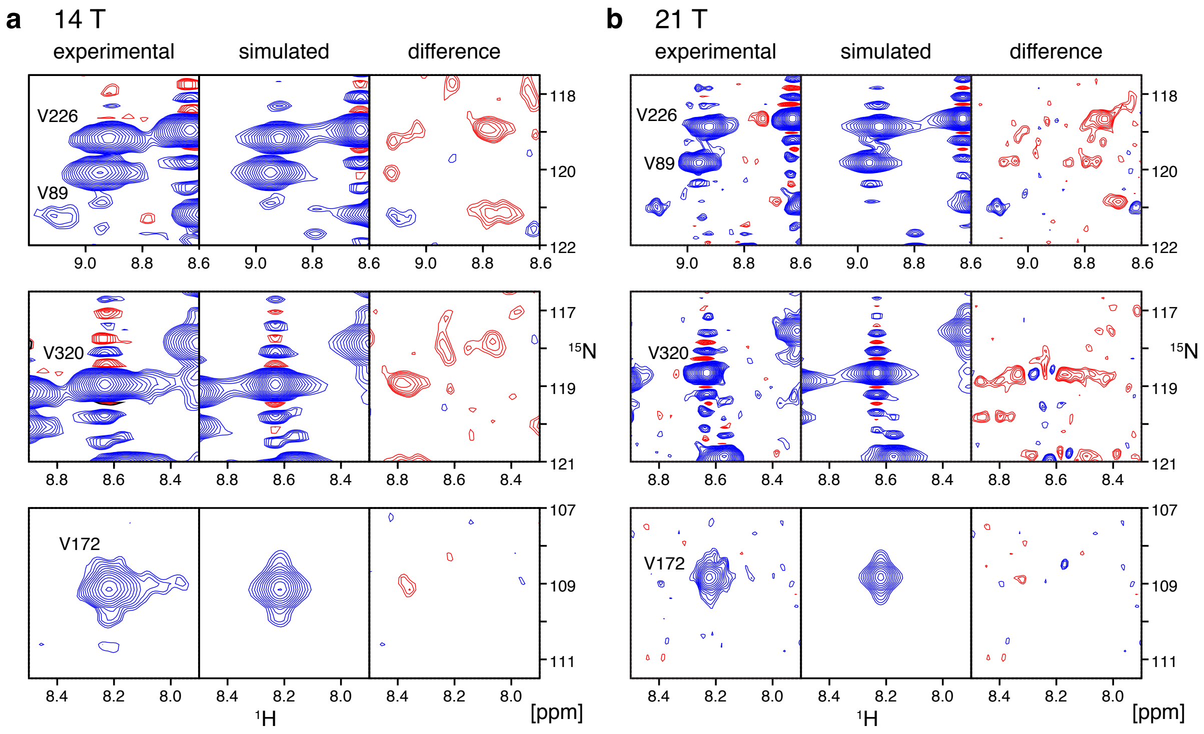

Supplementary Figure 4. Example of the quality of time-domain line shape fitting of ^{1}H-^{15}N correlation spectra. Selected regions of experimental, simulated and difference TROSY spectra of ^{15}N-valine labeled TS-β_1AR•alprenolol are shown for various resonances labeled with assignment information. (a) 14 T, (b) 21 T.
