# Supplementary material for: A high-resolution description of β1-adrenergic receptor functional dynamics and allosteric coupling from backbone NMR
Source: Nat Commun. 2020 May 5;11:2216. doi: 10.1038/s41467-020-15864-y (PMC7200737; doi:10.1038/s41467-020-15864-y)
Supplement: Supplementary file 2 — Description of Additional Supplementary Files [file 41467_2020_15864_MOESM2_ESM.pdf]

Description of Additional Supplementary Files

Supplementary Data 1: Pharmacological and geometrical properties of the β_1AR ligands

Supplementary Data 2: ^{1}H and ^{15}N chemical shifts of valine ^{15}N resonances of YY-β_1AR and TS-β1AR in various forms

Supplementary Data 3: ^{15}N relaxation rates of valine ^{15}N resonances of YY-β_1AR and TS-β1AR in various forms
